# Enhanced Expression of Trib3 during the Development of Myelin Breakdown in *dmy* Myelin Mutant Rats

**DOI:** 10.1371/journal.pone.0168250

**Published:** 2016-12-15

**Authors:** Yukako Shimotsuma, Miyuu Tanaka, Takeshi Izawa, Jyoji Yamate, Mitsuru Kuwamura

**Affiliations:** Laboratory of Veterinary Pathology, Osaka Prefecture University, Izumisano City, Osaka, Japan; Washington University, UNITED STATES

## Abstract

The demyelination (*dmy*) rat exhibits hind limb ataxia and severe myelin breakdown in the central nervous system. The causative gene of *dmy* rats is the MRS2 magnesium transporter gene. Tribbles homolog 3 (*Trib3*) is a pseudokinase molecule that modifies certain signal pathways, and its expression is increased in response to various stresses. Here we sought to clarify the mechanism of myelin breakdown by focusing *Trib3*, which is remarkably up-regulated in *dmy* rats. The expression of *Trib3* mRNA was significantly increased at 4, 5, 6, 7 and 8 weeks of age in the *dmy* rats, prior to the prominent myelin breakdown between 7 and 10 weeks of age. The expression level of *Trib3* was increased concurrently with the progression of the clinical and pathological conditions in the *dmy* rats. Double immunofluorescence demonstrated that TRIB3 was mainly expressed in neurons and oligodendrocytes and localized in the Golgi apparatus. Our findings indicate that *Trib3* may be associated with the pathogenic mechanism of *dmy* rats.

## Introduction

Myelin mutants are extremely useful as tools for clarifying the complex process of myelination, the maintenance of myelin, and myelin diseases. Many myelin mutants have been established, including the jimpy (*jp*) mouse [1] which has X-linked mutations affecting the proteolipid protein gene; the myelin-deficient (*md*) rat [2]; the shiverer (*shi*) mouse [3]; the Long Evans Shaker (*les*) rat [4], which has a genetic mutation in myelin basic protein and the *taiep* rat [5], which has a microtubule abnormality.

The demyelination (*dmy*) rat is a unique spontaneous myelin mutation that exhibits severe myelin breakdown with a late onset of clinical signs [6]. The causative autosomal recessive mutation has been identified at the MRS2 magnesium transporter (*Mrs2*) gene, which encodes an essential component of the major Mg^2+^ influx system in mitochondria; *Mrs2* gene is expressed mainly in neurons [7]. The *dmy* rat is characterized by severe myelin destruction throughout the white matter of the central nervous system (CNS) during the late stage of myelination, and the distribution progresses rapidly [8]. Hypertrophic oligodendrocytes were frequently observed in the ventral funiculus of the spinal cord of *dmy* rats, and the cytoplasm was intensely stained with mitochondrial markers [9]. These findings indicate that the *Mrs2* gene plays a prominent role in the development and maintenance of myelin. However, the detailed pathogenesis including the relationship between mitochondrial dysfunction and myelin disorder remains uncertain.

Tribbles homolog 3 (*Trib3*) has been reported as a pseudokinase with scaffold-like regulatory functions related to inflammation and certain signaling. *Trib3* expression is increased in response to various stresses, such as nutritional deficiencies [10, 11], endoplasmic reticulum stress [12–15], hypoxia [16] and oxidant stress [17]. *Trib3* has also been reported to be an important regulatory protein involved in signal pathways; for example, the inhibition of mitosis [18], the inhibition of insulin signaling by binding directly to Akt [19], the modulation of mitogen-activated protein kinase activity [20] and more. However, there are few reports describing the function of Trib3 in the CNS [21, 22]. To address the detailed pathomechanism of myelin destruction in *dmy* rats, we performed a microarray analysis using spinal cord samples and observed a remarkably up-regulated expression of *Trib3* gene. The purpose of this study is to shed light on the mechanism of myelin destruction in the *dmy* rat by focusing on the expression of Trib3.

## Materials and Methods

### Animals

The *dmy* rats were supplied by the National BioResource Project—Rat, Kyoto University (Kyoto, Japan). The rats were maintained at the Education and Research Center for Experimental Animal Science at Osaka Prefecture University. We mated heterozygous (*dmy*/+) females with heterozygous (*dmy*/+) males and obtained the affected (*dmy*/*dmy*) and control (+/+ or *dmy*/+) rats. We examined these rats at 4, 5, 6, 7 and 8 weeks of age.

For the comparison of *Trib3* expression in myelin lesions, we also examined two other myelin mutant rats, myelin vacuolation (*mv*) and vacuole formation (VF) rats. The *mv* rat is a spontaneous tremor mutant with a null mutation in the attractin gene; these rats show hypomyelination and vacuole formation in the myelin throughout the CNS from the early stage of myelination [23]. The VF rat shows tremor behavior (especially in the caudal body) from the age of approx. 10 days. The peak of tremor is observed around 4 weeks of age, and then, tremor behavior gradually improves [24]. Hypomyelination and abnormal vacuoles around the axons in VF rats were observed mainly in the white matter of the spinal cord, and the vacuoles were gradually reduced [24]. The VF rat has a mutation in *Dopey1* gene, which is likely to be involved in the traffic of myelin proteins [25]. We mated heterozygous (*mv*/+, *vf*/+) females with heterozygous (*mv*/+) or homozygous (*vf*/*vf*) males and obtained the affected (*mv*/*mv*, *vf*/*vf*) and control (+/+ or *mv*/+, *vf*/+) rats. As soon as the rats were weaned, we carried out genotyping by using the Amp-FTA method as described [26]. In the present study, we examined the affected homozygous and wild type control rats of *mv* and VF mutants at 4 and 6 weeks of age and at 4, 10, 20 weeks of age, respectively.

All of the rats were maintained in a room with controlled temperature and a 12-hr light-dark cycle. Food and water were provided *ad libitum*. This study was carried out in strict accordance with the recommendations in the Guidelines for Animal Experimentation of Osaka Prefecture University. The protocol was approved by the Animal Experimentation Committee of Osaka Prefecture University (Permit nos. 25–68 and 27–54). All rats used in this study were deeply anesthetized with isoflurane and exsanguinated from abdominal aorta, and all efforts were made to minimize suffering.

### Microarray analysis

For the microarray analysis, 6-week-old control and homozygous rats of each mutant were deeply anesthetized and exsanguinated from the abdominal aorta. Total RNA was isolated from the cervical spinal cord with the SV Total RNA isolation system (Promega, Madison, WI, USA). The quality of the RNA was checked with Lab Chips (Agilent technologies, Santa Clara, CA) on an Agilent 2100 Bioanalyzer (Agilent Technologies) and NanoDrop spectrophotometer ND-1000 (nanoDrop Technologies, Wilmington, DE). The RNA was then prepared and hybridized to the rat Genome 230 2.0 Array (Affymetrix, Santa Clara, CA) by Bio Matrix Research, Inc. (Nagareyama, Japan.), following the protocols provided by Affymetrix. The data analysis was done using Gene-Spring GX software [26].

### Real-time RT-PCR

Cervical spinal cords from *dmy* homozygous and control rats at 4, 5, 6, 7 and 8 weeks of age were dissected into the ventral funiculus, dorsal funiculus and gray matter. The *mv* and control rats at 4 and 6 weeks old and VF and control rats at 4, 10 and 20 weeks old were also necropsied, and the cervical spinal cords were dissected into the white and gray matter. Total RNA was isolated with the SV Total RNA isolation system (Promega) according to the manufacturer's instructions. A real-time PCR was performed with SYBR Green Real-time PCR Master Mix (Toyobo, Osaka, Japan) with the following setting: 40 cycles of amplification, 95°C for 5 s, 60°C for 30 s. The data were normalized to β-actin mRNA using the following primer pairs: Trib3 forward 5’-TCATCTTGCGCGACCTCA-3’ and reverse 5’-GTCCAGTCATCACACAG GCATC-3’; β-actin forward 5’-TAAAGACCTCTATGCCAACAC-3’ and reverse 5’-CTCCTGCTTGCTGATCCACAT-3’. The relative expression levels were calculated using the comparative Ct (threshold cycle) method.

### Immunohistochemistry

Lumbar spinal cords from 7-week-old *dmy* and control rats were removed and frozen at −80°C. Ten-μm frozen sections were cut using a cryostat. After drying, the sections were fixed in diethyl ether at 4°C for 10 min. The tissues were then rinsed in phosphate-buffered saline (PBS) and treated with 10% normal goat serum in PBS for 30 min. The sections were then incubated with mouse anti-TRIB3 antibody (clone 1H2) (1:100; Sigma-Aldrich, St. Louis, MO) at 4°C overnight. For the detection of Golgi apparatus, *dmy* and control rats were perfused transcardially with 4% paraformaldehyde (PFA) in 0.1 M phosphate buffer (PB; pH 7.4). Tissue samples from lumbar spinal cords were routinely processed and embedded in paraffin. Four-μm sections were dewaxed, pretreated in a microwave with citrate buffer (pH 6.0, 10 mM) for 20 min, and incubated in 5% skim milk for 30 min. The sections were then incubated overnight at 4°C with anti-Golgi 58K mouse monoclonal antibody (1:1000; Sigma-Aldrich). After incubation with the primary antibody, the sections were treated with 0.3% hydrogen peroxide/0.065% sodium azide in PBS to quench endogenous peroxidase activity. The sections were then treated with a peroxidase-conjugated anti-mouse IgG antibody (Histofine Simplestain MAX PO; Nichirei, Tokyo) for 60 min. Signals were visualized with a 3, 3’-diaminobenzidine (DAB) substrate kit (Nichirei).

For the immunofluorescence analysis, sections were rinsed in PBS containing 0.3% Triton X-100 (Sigma-Aldrich), and then treated with 10% normal goat serum in PBS for 30 min. The sections were then incubated with mouse anti-TRIB3 antibody at 4°C overnight and treated with the Alexa Fluor 488-labeled secondary antibody against mouse IgG (1:1000; Invitrogen, Carlsbad, CA) for 45 min and coverslipped by mounting medium with DAPI (Vector Laboratories, Burlingame, CA). Signals were detected with a confocal imaging system C1Si (Nikon, Tokyo).

For double immunofluorescence, sections were incubated with the combination of the following antibodies: polyclonal rabbit anti-GFAP for astrocytes (1:1000; Dako, Glostrup, Denmark), polyclonal rabbit anti-Olig2 for oligodendrocytes (1:1000; Immuno-Biological Laboratories, Gunma, Japan), monoclonal mouse anti-CD11b for microglia (1:200; AbD Serotec, Oxford, UK), polyclonal rabbit anti-SATB2 for neurons (1:500; Abcam, Cambridge, UK) and polyclonal rabbit anti-GM130 for Golgi apparatus (C-terminal) (1:2000; Sigma-Aldrich) antibodies. The sections were then incubated with secondary antibodies conjugated with Alexa 488 or 568 (Invitrogen) for 45 min, and coverslipped with DAPI mounting medium (Vector Laboratories). Signals were detected with a confocal imaging system (C1Si; Nikon).

### Statistical analysis

The data are presented as means ± standard deviation. The statistical analysis was performed using Tukey-Kramer tests. A *p*–value <0.05 was considered significant.

## Results

### *Trib3* mRNA expression was significantly increased in the *dmy* rats but not in the other myelin mutant rats

**Fig 1:**
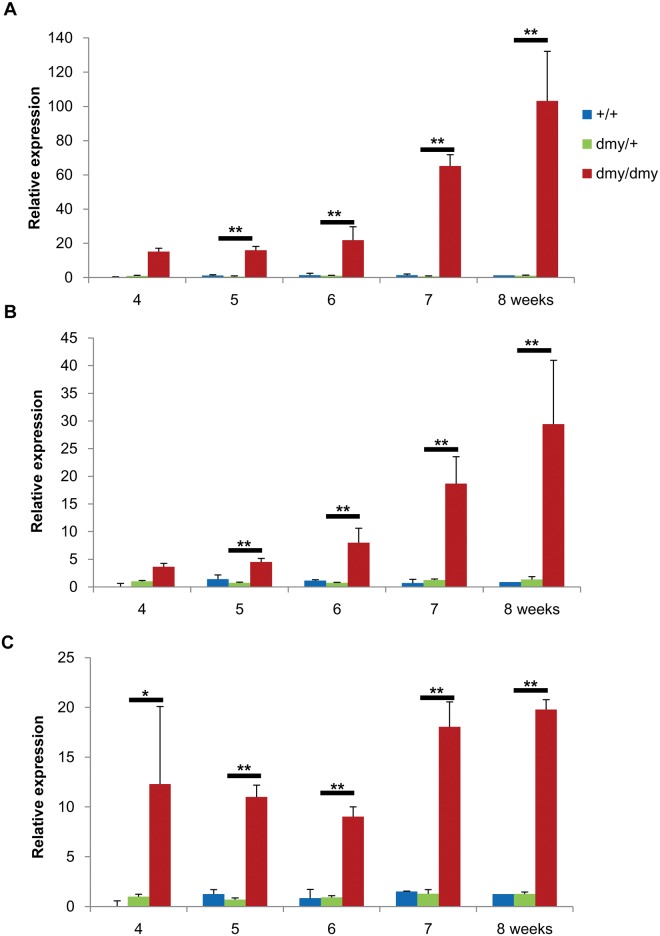
Real-time PCR for *Trib3*. Expression of *Trib3* gene in the ventral funiculus in the *dmy* rats (A), gray matter (B) and dorsal funiculus (C) of the cervical spinal cords from the control (+/+ and *dmy*/+) and the *dmy* (*dmy*/*dmy*) rats. The data are presented as the mean ratio of target to reference gene. β-actin is used as an internal control. In the *dmy* rats, expression levels of *Trib3* mRNA is significantly increased in all areas at 4, 5, 6, 7 and 8 weeks of age. *, *p* <0.05, **, *p* <0.01, Tukey-Kramer test (*n* = 3 in each group).

**Fig 2:**
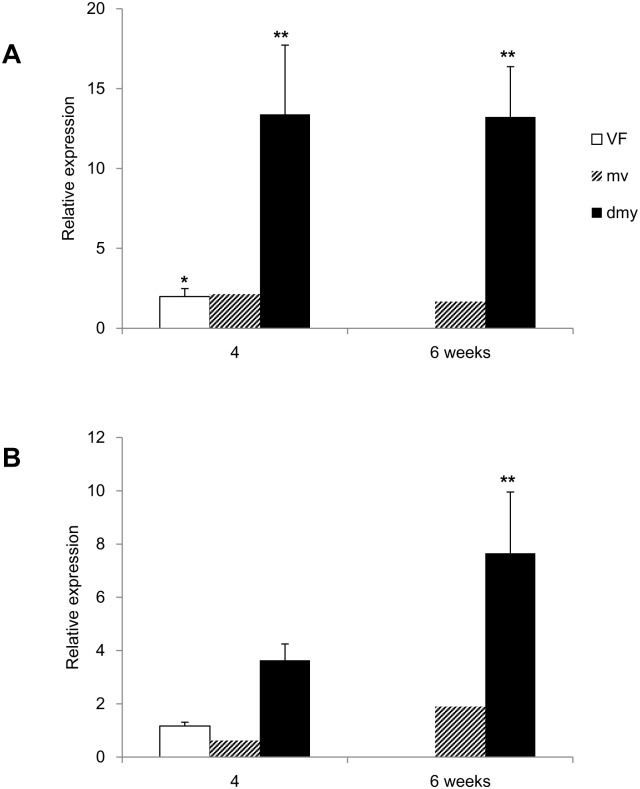
Real-time PCR for *Trib3* (VF, *mv* and *dmy* rats). Relative expression of *Trib3* in the white matter (A) and the gray matter (B) of the cervical spinal cords from the *dmy*, *mv* and VF rats by the real-time PCR. The data are presented as the mean ratio against expression in the control rats. Although expression levels of *Trib3* mRNA is significantly increased in the *dmy* rats, no significant differences are seen in the VF and *mv* rats. *, *p* <0.05, **, *p* <0.01, Tukey-Kramer test (*n* = 3 in VF and *dmy* rats. *n* = 2 in *mv* rats.).

The microarray analysis revealed a 6.2-fold higher expression of *Trib3* in the *dmy* rats compared to the control rats (Gene Expression Omnibus, accession no. GSE88855). We next investigated the *Trib3* mRNA expression in more detail by the real-time PCR analysis. The expression of Trib3 mRNA in the *dmy* rat was significantly increased in all areas of the spinal cord (i.e. the ventral funiculus, dorsal funiculus and gray matter) at all time points examined (4, 5, 6, 7 and 8 weeks); the expression in the ventral funiculus was the most prominent (Fig 1). We also observed that the increased expression became conspicuous with age in the ventral funiculus and gray matter. For the comparison of *Trib3* expression in the myelin lesions of the other myelin mutants, we also conducted a real-time PCR analysis in the *mv* and VF rats using samples from the white and gray matter of the spinal cord. The expression level of *Trib3* of the *dmy* rats was approx. 12-hold higher in the white matter compared to that in the controls at 4 and 6 weeks of age, and approx. 8-fold higher in the gray matter at 6 weeks of age (Fig 2). In the white matter of the VF rats at 4 weeks of age, a significant increase in the expression level of *Trib3* was seen; however, it was not as remarkable as that of the *dmy* rats (Fig 2A). No significant differences in the relative expression levels of *Trib3* were detected in the gray matter between the *mv* and VF rats (Fig 2B).

### Distribution and subcellular localization of TRIB3 in the *dmy* rat

**Fig 3:**
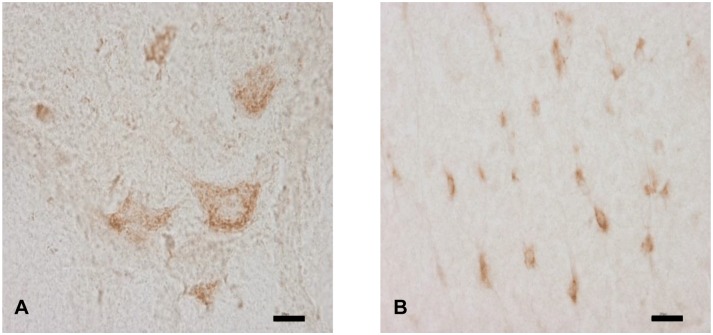
Immunohistochemistry for TRIB3 in the *dmy* rat. The immunohistochemistry for TRIB3 in the lumbar spinal cords from *dmy* rats at 7-week-old. Cytoplasm of neurons and glial cells are stained with TRIB3 in the gray (A) and white matter (B) of the *dmy* rats. Bars: 20 μm.

**Fig 4:**
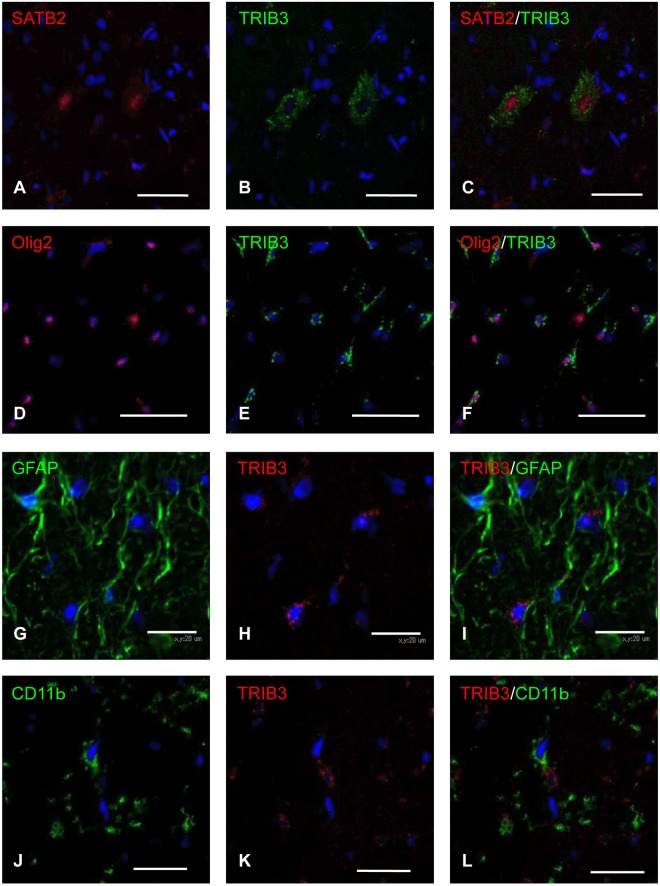
Double immunofluorescence for SATB2, Olig2, GFAP, CD11b and TRIB3. The double immunohistochemistry for SATB2 (A-C), Olig2 (D-F), GFAP (G-I), CD11b (J-L) and TRIB3 in the lumbar spinal cords from the *dmy* rats at 7-week-old. SATB2-positive neurons and Olig2-positive oligodendrocytes are positive for TRIB3, although GFAP-positive astrocytes and CD11b-positive microglial cells are not positive for TRIB3. Bars: A-F, J-L; 20 μm, G-I; 50 μm.

**Fig 5:**
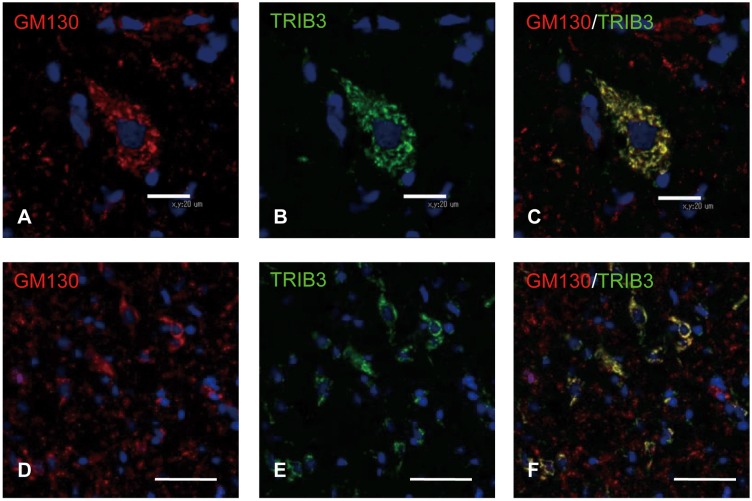
Double immunofluorescence for GM130 and TRIB3. The double immunohistochemistry for GM130 and TRIB3 in the gray matter (A-C) and white matter (D-F) of the lumbar spinal cords from the *dmy* rats at 7-week-old. There are many GM130/TRIB3 double positive cells in the gray matter (A-C) and white matter (D-F) of the *dmy* rats. Bars: 20 μm (A-C), 50 μm (D-F).

**Fig 6:**
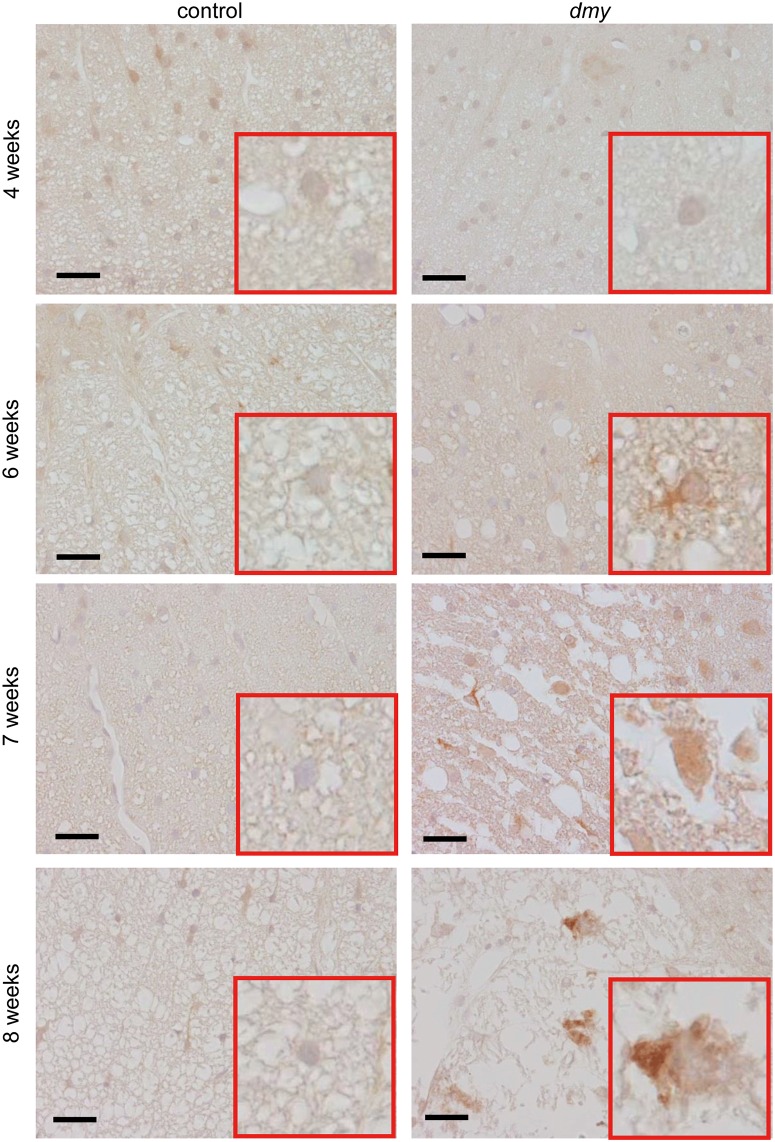
Immunohistochemistry for Golgi 58K. The immunohistochemistry for Golgi 58K in the lumbar spinal cords from the *dmy* rats at 4, 6, 7 and 8 weeks of age. The immunoreactivity for the Golgi apparatus is elevated in the ventral funiculus of the *dmy* rats at 6–8 weeks of age. Bars: 20 μm.

To identify the localization of TRIB3 in the spinal cord of the *dmy* rats, we conducted immunohistochemistry for TRIB3 in 7-week-old rats. The cytoplasm of the neurons and glial cells in the white matter were positively stained for TRIB3 in a dot-like pattern (Fig 3). To identify the TRIB3-positive cells in the *dmy* rats, we performed double immunofluorescence using specific markers for neurons, oligodendrocytes, microglial cells and astrocytes. TRIB3 was coexpressed with neurons and some of the oligodendrocytes, but not with microglial cells or astrocytes (Fig 4). We investigated the subcellular localization of TRIB3 by double immunofluorescence. It revealed the co-localization of TRIB3 and Golgi apparatus in neurons and oligodendrocytes (Fig 5). The immunohistochemistry for the Golgi apparatus marker revealed that immunoreactivity was elevated in the ventral funiculus of the *dmy* rats at 6–8 weeks of age (Fig 6).

## Discussion

Using a microarray analysis and real-time PCR, we observed the enhanced expression of *Trib3* mRNA in the *dmy* rats but not in the *mv* or VF rats. Elevated expression of *Trib3* in the *dmy* rats was observed especially in the ventral funiculus and gray matter. Particularly pronounced myelin lesions are observed in the ventral and lateral parts of the spinal cord in *dmy* rats at 7–10 weeks of age [8]. In agreement with a previous study’s finding that myelin lesions in *dmy* rats deteriorate with advancing age [8], we observed in the present study that the expression level of *Trib3* gradually increased from 4 to 8 weeks of age. Although no remarkable histopathological myelin-related lesions were found in the *dmy* rats up to 6 weeks old [8], in the present study we observed an increased level of *Trib3* expression in the 4–5 week-old rats, suggesting that *Trib3* may play some roles in the mechanism of myelin destruction in *dmy* rats from an early period.

The double immunofluorescence demonstrated that TRIB3 was expressed mainly in neurons and oligodendrocytes in the *dmy* rats. An up-regulated expression of TRIB3 may be related to a potential degeneration of neurons and/or oligodendrocytes in these rats. *Mrs2*, a causative gene for *dmy* rats encodes a component of the Mg^2+^ influx system in mitochondria, and in our analysis this gene was expressed mainly in neurons. No apparent alteration was found in neurons of the *dmy* rats, and the detailed mechanism underlying how dysfunction in mitochondria leads to the destruction of myelin remains unclear. Trib3 is induced in multiple cellular models of Parkinson’s disease (PD) and the dopaminergic neurons of human PD patients, and it is thus considered a potential mediator of neuronal death and degeneration in PD [27]. Oligodendrocytes are known to be a target of the autoimmune attack in demyelinating diseases such as multiple sclerosis (MS) and experimental autoimmune encephalomyelitis, an animal model of MS [28]. Moreover, oligodendrocytes in culture are more sensitive to oxidative stress exerted by H_2_O_2_ compared to astrocytes and microglial cells [29]. In the present study’s microarray analysis, the expression levels of hemeoxygenase-1 (HO-1) and NAD(P)H dehydrogenase, antioxidant enzymes, were increased in the *dmy* rats. This is consistent with previous studies’ findings that HO-1 was predominantly induced in oligodendrocytes during the early stage of demyelination in the *dmy* rat but not in the *mv* rat, and it suggests that oxidative stress is likely involved in the pathogenesis of demyelination in the *dmy* rat [30].

Our immunohistochemistry for Golgi 58K has demonstrated that immunoreactivity for the Golgi apparatus was elevated in the ventral funiculus in the *dmy* rats between 6 and 8 weeks of age. Our double immunofluorescence also demonstrated that TRIB3 is localized in the Golgi apparatus. Oxidative stress enhances C/EBP homologous protein (CHOP), a marker of endoplasmic reticulum (ER) stress, and increases the binding of CHOP to the Trib3 promoter in murine diabetic kidneys [31]. The overexpression of Trib3 is thus likely to be promoted by oxidative stress, and ER stress may be a possible reason for the myelin destruction in the *dmy* rat.

Several studies have shown that oxidative stress and ER stress induce the apoptosis of oligodendrocytes and suppress remyelination in demyelinating lesions in MS [29, 32–34]. Trib3 is considered to be a target of CHOP/ATF4 and works as a sensor for ER stress-induced apoptosis [14]. If the ER stress is transient and mild, the induced Trib3 blocks the CHOP and ATF4 function via a negative feedback mechanism and represses apoptosis. However, when the ER stress is potent and prolonged, excess Trib3 will be produced and lead to apoptosis by dephosphorylating Akt and inducing the translocation of Bax to mitochondria, which accelerates programmed cell death. Trib3 is also involved in apoptosis via Caspase 3 (CASP3) [35]. Caspases play a central role in the execution-phase of apoptosis, and Trib3 prevents the activation of CASP3 by the nuclear translocation of proCASP3. However, in conditions of prolonged stress, Trib3 is cleaved by CASP3 and could be a trigger for the further activation of caspases and induce apoptosis. This indicates that Trib3 functions as a molecular switch between the cell survival and apoptotic pathways under stressful conditions. Aime *et al*. considered that an elevated expression of Trib3 may enhance the proapoptotic activities and be related to the promotion of neuronal death in PD and its models [27]. Using a TUNEL assay and electron microscopy, we did not observe any apparent increased apoptosis in the spinal cord of the *dmy* rats at 7 weeks of age. However, *dmy* rats exhibit severe myelin destruction throughout the white matter of the CNS during the late stage of myelination, and it might thus be difficult to detect apoptotic cells. Further research is needed to understand the precise role of increased *Trib3* expression in the *dmy* rat.
